# Comparing the Effects of Collagen Hydrolysate and Dairy Protein on Recovery from Eccentric Exercise: A Double Blind, Placebo-Controlled Study

**DOI:** 10.3390/nu16244389

**Published:** 2024-12-20

**Authors:** Rachel Barclay, Jane Coad, Katie Schraders, Matthew J. Barnes

**Affiliations:** 1 School of Sport, Exercise & Nutrition, College of Health, Massey University, Palmerston North 4410, New Zealand; 2 School of Food Technology & Natural Sciences, College of Science, Massey University, Palmerston North 4410, New Zealand

**Keywords:** collagen hydrolysate, delayed-onset muscle soreness, exercise-induced muscle damage, post-exercise recovery, milk proteins

## Abstract

Background: Consuming collagen hydrolysate (CH) may improve symptoms of exercise-induced muscle damage (EIMD); however, its acute effects have not been compared to dairy protein (DP), the most commonly consumed form of protein supplement. Therefore, this study compared the effects of CH and DP on recovery from EIMD. Methods: Thirty-three males consumed either CH (*n* = 11) or DP (*n* = 11), containing 25 g of protein, or an isoenergetic placebo (*n* = 11) immediately post-exercise and once daily for three days. Indices of EIMD were measured before and 30 min and 24, 48, and 72 h after 30 min of downhill running on a −15% slope at 80% of VO_2max_ speed. Results: Downhill running induced significant EIMD, with time effects (all *p* < 0.001) for the delayed onset of muscle soreness (visual analogue scale), countermovement jump height, isometric midthigh pull force, maximal voluntary isometric contraction force, running economy, and biomarkers of muscle damage (creatine kinase) and inflammation (interleukin-6, high-sensitivity C-reactive protein). However, no group or interaction effects (all *p* > 0.05) were observed for any of the outcome measures. Conclusions: These findings suggest that the post-exercise consumption of CH or DP does not improve indices of EIMD during the acute recovery period in recreationally active males.

## 1. Introduction

Exercise-induced muscle damage (EIMD) is caused by exercise that is strenuous, repetitive, novel, and/or involves eccentric muscle contractions, such as downhill running [1]. Whilst EIMD is temporary, and may be beneficial for future muscle adaptation [2], it can create problems for regular exercisers by impairing performance during subsequent bouts of exercise [3] and/or by causing a disruption to normal daily activities that are not otherwise an issue [4]. Therefore, timely recovery from EIMD is important.

Nutritional supplements and functional foods may provide benefits for physical health [5], performance [6], and recovery from exercise [7]; however, although significant effort has been made to find nutritional interventions that alleviate the symptoms of EIMD, including omega-3 fatty acids, various amino acids, polyphenols, and herbal extracts, there is no strong consensus on the best option [8]. One intervention at the forefront of most recommendations is dairy protein (DP) [9], which includes whey, casein, and whole milk. The consumption of DP following EIMD is believed to improve recovery through the delivery of essential amino acids that enhance rates of muscle protein synthesis, thus promoting muscle repair [10]; however, this mechanism has been disputed [11]. Irrespective of the mechanism, whether consuming DP is beneficial for recovery remains unclear, with some studies showing that DP can alter markers of EIMD, including muscle function (for example, Brown et al. [12]; Buckley et al. [13]; Cooke et al. [14]), while others, including Gee et al. [15]; Nieman et al. [16]; Ormsbee et al. [17]; and Saracino et al. [18], have failed to see an effect. Moreover, the majority of research has shown that supplementing with protein, including DP, does not specifically reduce exercise-induced delayed-onset muscle soreness (DOMS) [19]. Despite mixed evidence for its effects on EIMD, and a significant environmental footprint [20], DP’s convenience and effects on muscle protein synthesis make it the major contributor to a protein supplement industry that is expected to be worth over USD 10 billion by 2030 in the US alone [21].

In order to improve sustainability, reduce environmental harm, and open up new commercial markets, researchers (and the supplement industry) are increasingly interested in novel protein sources [22] and how they may impact recovery and performance [19,21]. Sourced from the underutilised by-products of the meat industry, bovine collagen is high in the non-essential amino acids used to synthesise collagen and remodel the extra-cellular matrix (ECM) [23]. Once ingested, collagen hydrolysates (CHs) are digested in the gastrointestinal tract to yield free amino acids [24], as well as collagen-specific di- and tripeptides, including hydroxyprolylglycine (Hyp-Gly) and prolylhydroxyproline (Pro-Hyp) [25]. These are absorbed intact, via specific transporters on the enterocyte brush border membranes [26], and are increased in circulation following the ingestion of CH [25]. Additionally, Pro-Hyp is produced naturally as a product of collagen degradation in damaged tissues [27], where it stimulates the proliferation of fibroblasts and subsequent collagen synthesis; collagen-derived Pro-Hyp is therefore considered a fibroblast growth-initiating factor [28]. The effect of endogenous, wound-derived Pro-Hyp appears to be enhanced by Pro-Hyp from dietary CH [27]; therefore, CHs may offer a simple and inexpensive approach to optimising recovery from connective tissue damage, as occurs with EIMD [2].

Damage to the ECM negatively impacts force transmission [29], storage and utilisation of elastic energy during the stretch-shortening cycle [30], and, because of the high density of nociceptors located in the ECM, may be a major contributor to DOMS [31]. As such, enhancing the rate of repair is likely important for recovery. Indeed, there is some, limited, evidence showing that CH attenuates several markers of EIMD, most notably DOMS [32,33] and movements that rely on the stretch-shortening cycle [30,32]. Greater evidence for such benefits may provide economic opportunities and optimise the use of a food industry waste product, thus helping to improve the sustainability and environmental impact of the protein industry by reducing the reliance on DP. Although studies have compared the effects of CH and DP on rates of protein synthesis [34], and a combination of whey and CH on EIMD [9], to date, the separate effects of DP and CH have not been directly compared. A greater understanding of whether either protein source is better than the other, or indeed better than a placebo, may help inform consumers who are considering protein supplementation as an aid for muscle recovery. Therefore, we compared the effects of CH and DP on acute recovery from EIMD. It was hypothesised that, when consumed in the days after eccentric exercise, CH would reduce ratings of DOMS, increase the recovery of muscle function, and attenuate bloodborne biomarkers of muscle damage and inflammation following EIMD, to an equal or greater extent than an equivalent amount of protein from DP, and that both protein sources would be more effective than an isoenergetic placebo (PLA).

## 2. Materials and Methods

### 2.1. Participants

**Figure 1:**
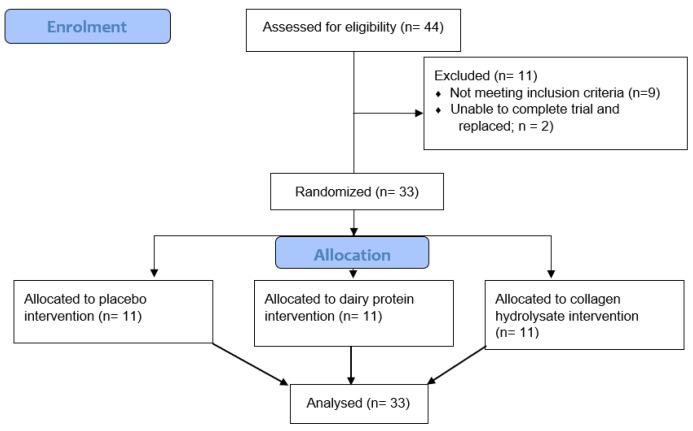
CONSORT flow diagram for recruitment and data collection.

Thirty-three active, healthy males aged between 18 and 40 years participated in this study. All participants performed aerobic and resistance exercise at least twice per week in the six months prior to this study and, based on their maximal oxygen uptake (VO_2max_; Table 1), had fair to good aerobic fitness [35]. Individuals who regularly consumed protein or other potentially ergogenic supplements (e.g., pre-workout products, branched-chain amino acids, or creatine monohydrate), consumed more than 1.6 g protein/kg body mass/day, who were vegan or vegetarian, and/or who were participating in other research studies were excluded. Participants completed a 24 h diet record one week before familiarisation to ascertain their habitual protein consumption, daily energy intake, and if they were consuming any ergogenic supplements. This was analysed by a nutritionist and was used to determine each participant’s suitability for participation. Potential participants who habitually consumed greater than 1.6 g protein/kg body mass/day and/or consumed ergogenic supplements were excluded from this study. If suitable, participants were asked to attend a familiarisation session in the Human Performance Laboratory, School of Sport, Exercise and Nutrition at Massey University, Palmerston North. The participants were instructed to maintain their habitual diet and refrain from strenuous exercise, alcohol, and anti-inflammatory drugs from 48 h before the main trial and for the duration of this study. All participants were volunteers, recruited by means of public advertisement. The recruitment and data collection process is outlined in Figure 1. Health screening and written informed consent were completed prior to the familiarisation. Ethical approval was obtained from the New Zealand Health and Disability Ethics Committee (HDEC EXP 12330), and this study was registered with the Australian and New Zealand Clinical Trials Register (ACTRN12622000529741).

### 2.2. Experimental Design

A double-blind, placebo controlled, between-subjects design was used to compare the effects of CH, DP, and PLA on recovery from 30 min of downhill running. Participants were stratified by VO_2max_ and then randomly assigned to one of three groups (CH, DP, PLA).

During the familiarisation session, participants completed a health screening questionnaire and provided informed written consent, before individual characteristics were collected (Table 1). They were then familiarised with the measures of muscle soreness, muscle function, and the muscle-damaging protocol (downhill running). They then completed a VO_2max_ test to establish running speeds for use in the main trial. Having had an opportunity to ask any further questions, they were then provided with a plastic shaker that contained 61.6 g (1000 kJ) of maltodextrin powder and were instructed to mix this with 250 mL of water and consume it two hours before returning to the laboratory for the main trial. This drink ensured that each participant consumed an equal amount of energy prior to the downhill run and, because each treatment contained 1000 kJ, that energy intake two hours before each measure (pre-downhill run (PRE), 24, 48, and 72 h post-downhill run) was the same.

At least one week after familiarisation, participants returned to the laboratory for the main trial at a convenient time for them. To understand and compare the effects of the interventions used in this study, and to allow for comparison with other similar studies [9,12,13,30,32,36,37,38,39,40,41,42,43], a series of commonly used, validated measures of EIMD were chosen [44]. PRE indices of EIMD were measured (blood sampling, soreness, muscle function, and running economy) and then participants completed 30 min of downhill running at 80% of their predetermined VO_2max_ speed. After a 30 min rest, indices of EIMD were measured again, and participants consumed their first supplement drink (CH, DP, or PLA).

For the three subsequent follow-up visits (24, 48, and 72 h post-downhill run), participants were instructed to consume their allocated supplement drink two hours before returning to the laboratory.

### 2.3. Supplements

To ensure double blinding, the supplement powders (CH (bovine collagen hydrolysate; FoodPilot, Massey University, New Zealand, and maltodextrin; Archer Daniels Midland Company, Chicago, IL, USA), DP (milk protein concentrate 470; Fonterra Cooperative Group Ltd., New Zealand, and maltodextrin), and PLA (maltodextrin)) were sealed in foil packaging and labelled A, B, or C by staff not involved in this study. Prototype spray-dried enzymatic CH was prepared conventionally from frozen, fresh dehaired cow hide in the FoodPilot, Massey University, Palmerston North, New Zealand. Hide was provided by Southern Pastures (Auckland, New Zealand ) Ltd. (New Zealand Business Number 9429031978873). While previous studies have used whey, casein, or whole milk, we used milk protein concentrate, which contains both whey and casein. It is believed that consuming this has a synergistic effect, where simultaneously there is an immediate effect from faster-acting whey protein and also a sustained effect from slower-acting casein protein [45]. All supplements were isoenergetic and flavour-matched with artificial and natural vanilla flavourings (see Table 2 and Table S1 for supplement details and amino acid profiles, respectively). The CH and DP supplements also contained an equal amount of protein (25 g [46]). Each serving was mixed in a plastic shaker with 250 mL of water and then consumed.

### 2.4. VO_2max_

A graded treadmill running test was used to determine participants’ oxygen consumption at four submaximal running speeds and to ascertain VO_2max_ (L/min). Participants were fitted with a silicone mask that covered their nose and mouth, and a heart rate strap (Polar Electro Oy, Kempele, Finland) was fitted around the chest. Breath-by-breath gas analysis was performed, and heart rate was measured continuously throughout the test using a metabolic cart (Quark CPET, Cosmed, Rome, Italy). Participants ran continuously on a motorised treadmill (True, St Louis, MO, USA) at 1% incline for four increasingly faster, predetermined, submaximal speeds for four minutes per stage. After the last submaximal stage, the speed was increased by 1 km/hour every minute until VO_2max_ was achieved. The relationship between submaximal oxygen consumption and running speed was used to formulate a linear regression equation. This equation, in conjunction with VO_2max_, was used to calculate running speed at 60% and 80% VO_2max_ for each participant.

### 2.5. Muscle-Damaging Exercise Protocol

Previous studies investigating the effects of protein supplementation on recovery have used a range of exercise modalities, including maximal eccentric knee extensions [13,18,38,39,43,47], drop jumps [9,30,32,36,41,48], various resistance exercise protocols [10,14,17,33,34,40,42], and downhill running [16,49]. As such, there is no standard methodology used to experimentally cause EIMD. In order to minimise participant burden and avoid the repeated bout effect [50], we asked participants to complete a single bout of downhill running. Bontemps et al. [51] suggest that downhill running has direct applicability to real-world exercise scenarios, such as off-road/trail and on-road running, and that it is appropriate for inducing EIMD in participants who are unfamiliar with this mode of exercise, as was the case with our participants. Additionally, downhill running has been shown to specifically alter the measures used in our study [51].

Muscle damage was induced using a modified downhill running protocol that has successfully been used and validated in similar studies [52,53]. Participants ran at 80% of their predetermined VO_2max_ speed on a motorised treadmill (VacuMed, Ventura, CA, USA) set at −15% incline for 30 min.

### 2.6. Muscle Soreness

A visual analogue scale (VAS) was used to assess participants’ self-reported muscle soreness. After holding a bodyweight squat for three seconds at a 90° knee angle, the participants were asked to rate the muscle soreness of their lower body on a scale of 0–10 (0 being no soreness and 10 being extreme soreness) by marking a vertical line through a 100 mm horizontal line [41]. Using the same VAS, the participants also rated the muscle soreness of their right quadriceps after performing three maximal voluntary isometric contractions (MVICs) of the right knee extensors [39]. The distance from zero on the VAS was recorded and compared between time points.

### 2.7. Muscle Function Measures

#### 2.7.1. Counter Movement Jump

Counter movement jump (CMJ) height (cm) was measured using a digital jump mat (SmartSpeed, Milton, QLD, Australia). Participants stood on the mat, placed their hands on their hips, and used a counter movement to perform a maximal vertical jump without any tucking of the knees while in flight [30]. Three CMJ attempts were made, with 30 s rest in between jumps. The highest jump was used for the analysis.

#### 2.7.2. Isometric Midthigh Pull

The peak isometric force of the lower body was determined using an isometric midthigh pull (IMTP [54]). Participants stood on a custom-made platform, bending the hips and knees to 120° and 140°, respectively. Measured with a goniometer at each time point, these joint angles ensured that the bar was positioned at approximately midthigh level [55]. The bar was connected to the platform and a load cell via a chain. After a verbal countdown, participants were told to produce maximal force for three seconds by attempting to extend at the hips and knees. Peak force (N) was then recorded, with the best out of three attempts used for the analysis.

#### 2.7.3. MVIC

MVIC of the right knee extensors was assessed using a custom-made isometric dynamometer. Participants were seated upright with their hips and knees at 90° flexion and a seat belt was fastened across their lap. A strap was also placed around their right ankle, securing them to a lever arm that was attached to a load cell. The load cell was connected to a custom-made amplifier and data were recorded using a Powerlab data acquisition unit (ADInstruments, Bella Vista, NSW, Australia) with force (N) recorded in Chart for Windows (v8, ADInstruments, Australia). After being strapped in place, a three second countdown was given, and the participant attempted to maximally extend their right knee for three seconds [56]. They repeated this three times, with 30 s rest between attempts. The highest peak force was recorded and used for the analysis.

### 2.8. Running Economy

Participants warmed up for five minutes on a motorised treadmill, set at a 1% incline, at 60% of their previously determined VO_2max_ speed. The speed was then increased to 80% of VO_2max_ speed, an intensity that has been shown to be impacted after downhill running [52], for an additional five minutes. Respiratory gases were sampled and analysed continuously and VO_2_ (L/min) was averaged over the last minute and used for the analysis.

### 2.9. Blood-Borne Biomarkers

Blood samples were drawn at PRE and 30 min, 24, 48, and 72 h post-downhill run from an antecubital vein by a trained phlebotomist and collected into vacutainer tubes (24 mL total per draw). They were then centrifuged and stored at −80 °C until analysis. The samples were analysed by Canterbury Health Laboratories (Christchurch, New Zealand) for creatine kinase (CK), high-sensitivity C-reactive protein (hsCRP), and interleukin-6 (IL-6). Plasma CK and hsCRP were determined on a AU5822 Clinical Chemistry Analyser (Beckman Coulter Inc., Brea, CA, USA). IL-6 was analysed using an ELISA (Invitrogen^TM^, Thermo Fisher Scientific, Waltham, MA, USA).

### 2.10. Statistical Analysis

Sample size for the repeated-measures design was calculated using G*Power software (version 3.1.97; Heinrich-Heine-Universität Düsseldorf, Düsseldorf, Germany). Using 80% power, moderate effect size, and an alpha of 5%, a total of *n* = 9 participants per group were needed to determine significant differences in responses between groups. However, to account for participant drop out and/or non-compliance, this study aimed to recruit 33 participants as a minimum and 36 participants as a maximum.

All analysis was performed in SPSS (version 28.0.1.1 SPSS Inc., Chicago, IL, USA). Baseline participant characteristics were compared using a one-way analysis of variance (ANOVA). Prior to the analysis of the results, data were examined for normality using the Shapiro–Wilk test. Non-normal data (CK, hsCRP, and IL-6) were nlog transformed prior to the analysis. Mauchley’s test was used to assess sphericity (ε) and, where the assumption of sphericity was violated, adjustments to the degrees of freedom were made (ε > 0.75 = Huynh–Feldt; ε < 0.75 = Greenhouse–Geisser). After examining for normality, a two-factor mixed ANOVA with repeated measures was used to identify differences between treatments (DP, PLA, or CH), time (pre and 30 min and 24, 48, and 72 h post), and the treatment × time interaction. Where main or interaction effects were identified, post hoc analysis using the Bonferroni adjustment was carried out. Partial eta squared was used to determine the effect size (small effect: ηp^2^ ≥ 0.01, medium effect: ηp^2^ ≥ 0.06, large effect: >0.14). Statistical significance was set to *p* < 0.05. All data are reported as mean ± SD.

## 3. Results

### 3.1. Participant Characteristics

No differences in mean age, height, body mass, aerobic fitness (VO_2max_), downhill running speed, and energy and protein intake between treatment groups were found (all *p* > 0.35; Table 1).

### 3.2. Muscle Soreness

**Figure 2:**
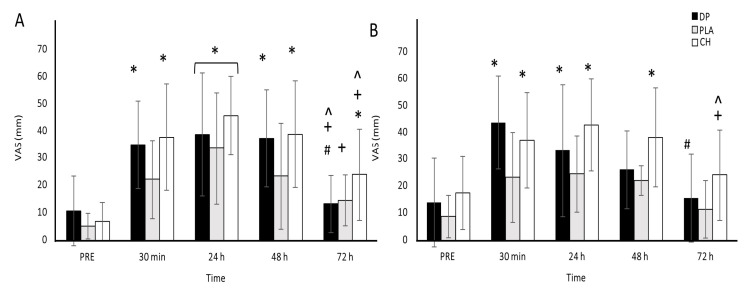
Muscle soreness, measured on a 100 mm visual analogue scale (VAS), during a squat (**A**) and after three MVICs (**B**) before (PRE) and after 30 min of downhill running. Participants were allocated into dairy protein (*n* = 11, DP), placebo (*n* = 11, PLA), or collagen hydrolysate (*n* = 11, CH) groups. * Different to PRE (*p <* 0.05); # different to 30 min (*p <* 0.05); + different to 24h (*p <* 0.05); ^ different to 48 h (*p <* 0.05).

Muscle soreness increased following downhill running across all treatments (Figure 2). Large significant time effects for muscle soreness were observed during the squat (*p* ≤ 0.001, ηp^2^ = 0.514) and during the MVIC (*p* #x2264; 0.001, ηp^2^ = 0.424). However, no significant treatment effect for muscle soreness was observed for either measure (during the squat: *p* = 0.063, ηp^2^ = 0.168; during the MVIC: *p* = 0.065, ηp^2^ = 0.189) and, similarly, no treatment x time interaction effects were found during the squat (*p* = 0.401, ηp^2^ = 0.066) or during the MVIC (*p* = 0.225, ηp^2^ = 0.94).

### 3.3. Muscle Function

**Figure 3:**
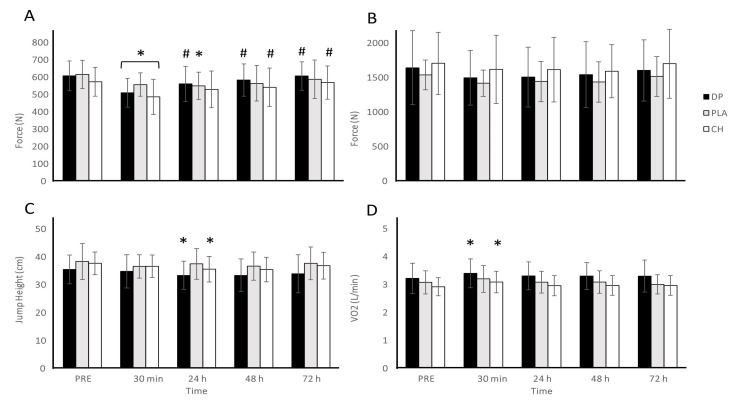
MVIC (**A**), IMTP (**B**), CMJ (**C**), and running economy (**D**) before (PRE) and after 30 min of downhill running. Participants were allocated into dairy protein (*n* = 11, DP), placebo (*n* = 11, PLA), or collagen hydrolysate (*n* = 11, CH) groups. * Different to PRE (*p <* 0.05); # different to 30 min (*p <* 0.05).

All measures of muscle function were reduced over time after completion of the downhill run (Figure 3). Large significant time effects were observed across all measures of muscle function (CMJ: *p* #x2264; 0.001, ηp^2^ = 0.182; IMTP: *p* #x2264; 0.001, ηp^2^ = 0.180; MVIC: *p* #x2264; 0.001, ηp^2^ = 0.430; running economy: *p* #x2264; 0.001, ηp^2^ = 0.229). Although time effects were observed, we did not find differences between treatments (CMJ: *p* = 0.332, ηp^2^ = 0.71; IMTP: *p* = 0.574, ηp^2^ = 0.036; MVIC: *p* = 0.559, ηp^2^ = 0.038; running economy: *p* = 0.153, ηp^2^ = 0.121), and no significant interaction effects were found (CMJ: *p* = 0.493, ηp^2^ = 0.059; IMPT: *p* = 0.994, ηp^2^ = 0.011; MVIC: *p* = 0.139, ηp^2^ = 0.095; running economy *p* = 0.978, ηp^2^ = 0.017).

### 3.4. Blood-Borne Markers

**Figure 4:**
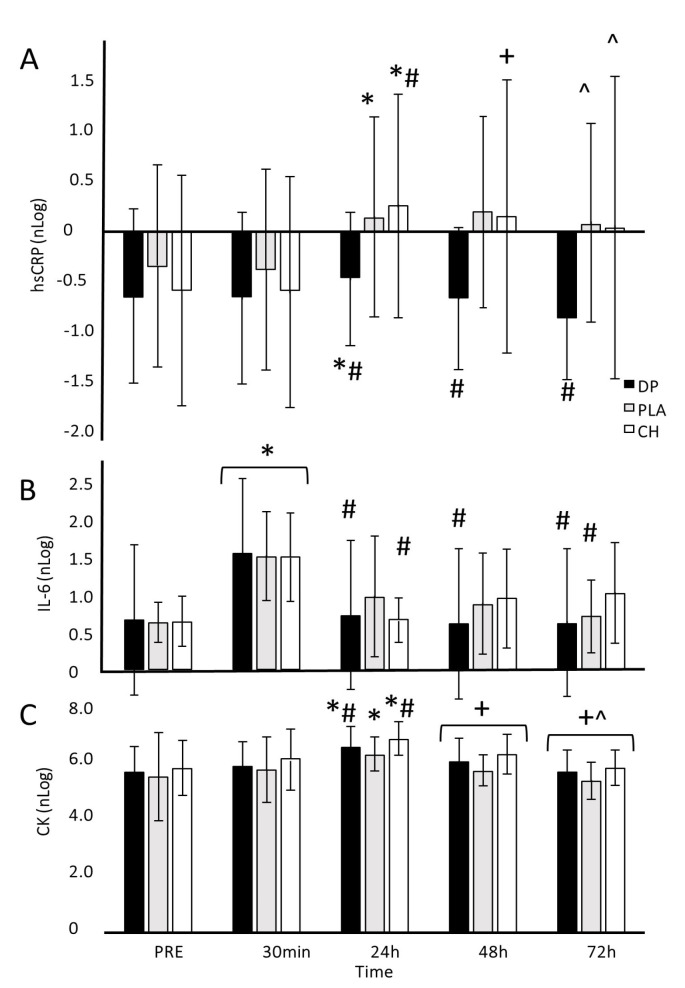
nLog of high-sensitivity C-reactive protein (hsCRP; (**A**)), interleukin 6 (IL-6; (**B**)), and creatine kinase (CK; (**C**)) before (PRE) and after 30 min of downhill running. Participants were allocated into dairy protein (*n* = 11, DP), placebo (*n* = 11, PLA), or collagen hydrolysate (*n* = 11, CH) groups. * Different to PRE (*p <* 0.05); # different to 30 min (*p <* 0.05); + different to 24 h (*p <* 0.05); ^ different to 48 h (*p <* 0.05).

There were large time effects for nlog hsCRP (*p* = 0.003, ηp^2^ = 0.143), nlog CK (*p* #x2264; 0.001, ηp^2^ = 0.531), and nLog IL-6 (*p* = 0.013, ηp^2^ = 0.48) (Figure 4). However, no treatment (hsCRP *p* = 0.318, ηp^2^ = 0.084; CK *p* = 0.488, ηp^2^ = 0.054; IL-6 *p* = 0.226, ηp^2^ = 0.018) or interaction effects (hsCRP *p* = 0.341, ηp^2^ = 0.081; CK *p* = 0.966, ηp^2^ = 0.022; IL-6 *p* = 0.261, ηp^2^ = 0.091) were found. CK and hsCRP peaked 24 h post-exercise before returning towards baseline levels at 72 h. IL-6 peaked 30 min after exercise; no other time points were significantly different to the pre-exercise values.

## 4. Discussion

EIMD has the potential to reduce performance during subsequent bouts of exercise and/or cause a disruption to normal daily activities [3,4]. According to Robberechts et al. [9], DP has been the standard nutrition intervention used to expedite recovery and minimise the effects of EIMD, and there is a modest body of evidence to suggest that it is beneficial in this context [2]. Additionally, there is growing evidence to suggest that CH may offer similar benefits [30,32,33,57]. However, to the authors’ knowledge, at this time, no one has compared the effects of consuming the two protein sources in the days after damaging exercise. Therefore, this study induced muscle damage to the lower limbs, using downhill running, in order to compare the effects of CH, DP, and an isoenergetic PLA on indices of EIMD up to 72 h post-exercise. All of the indices of EIMD significantly changed over time as a result of 30 min of downhill running, suggesting that muscle damage had occurred. However, in contrast to our hypothesis, CH was not equal, nor more beneficial than DP, and neither protein source was better than the PLA.

As expected, the damaging protocol used in our study resulted in large significant changes in muscle function, soreness, and blood-borne markers of EIMD over time. However, decrements in muscle function were mostly mild with significant changes in running economy and CMJ limited to 30 min and 24 h post-exercise, respectively, while no significant changes in IMTP were evident at any time point for any treatment. Decreases in MVIC force occurred with each treatment, albeit at different times and, again, to a mild level [58]. Similarly, measures of soreness changed after exercise, with the lowest change occurring with PLA. However, despite treatment effects approaching significance, to say that PLA was more beneficial than either protein source would be speculative. As reported elsewhere, CK [59] and hsCRP [60] peaked at 24 h, before returning towards baseline in the days after exercise, for all treatments. IL-6 increased in response to exercise [61] but does not appear to have been elevated by subsequent muscle damage. Collectively, these results suggest that 30 min of downhill running induced a mild to moderate amount of muscle damage.

The proposed mechanisms responsible for any benefit of DP or CH on recovery are theoretically sound, in that DP provides amino acids for the repair and synthesis of skeletal muscle [10,34] and CH provides amino acids and peptides, in particular Pro-Hyp, for the repair of connective tissue [28]. Additionally, both protein sources have anti-inflammatory actions [47,62], which may further benefit recovery. However, despite this, the outcomes of EIMD studies are inconsistent. DP, in its various forms, consumed in the days after damaging exercise has been shown to benefit the recovery of muscle function [12,13,14,38,49]; however, many more studies have failed to see an effect [15,16,17,18,36,37,40,41,43,63,64,65]. The same is true in regard to DOMS, with only a small number of studies reporting that DP can attenuate acute muscle soreness [39,42,47]. In fact, the majority of studies have failed to observe a benefit [12,13,15,16,17,18,36,37,38,40,41,43,49,63,64,65,66]. Further, of the studies mentioned, none found an effect of DP on inflammation, and only five saw an effect on the bloodborne markers of muscle damage [12,16,38,39,42]. In line with this overwhelming evidence, and therefore perhaps not surprising, we also failed to see an effect of DP on any indices of EIMD at any time point of recovery.

In the studies specifically investigating the influence of CH on EIMD, a positive effect on muscle function has been shown, but only during the counter movement jump [30,32]; a benefit to maximal isometric force recovery has not been observed. Given the potential effect of CH on the ECM, we expected to see some benefit on DOMS [32,33] and muscle performance [30,32]; in particular the counter movement jump and running economy, which both rely on the stretch-shortening cycle. However, this was not a given as the results of previous studies are inconsistent [9,30,32,33,57]. The disparate findings may in part be due to the various issues that plague many protein intervention studies [67]. These include, but are not limited to, different modes of exercise used to induce, and criteria used to assess, EIMD; dosing, timing, and duration of interventions; participant training history; choice of control/placebo [9,38,39,42]; insufficient levels of damage; studies being under powered [18,30,66]; and, perhaps most importantly [65], a lack of control over dietary protein intake [10,15,16,17,36,38,41,64].

Most studies investigating the effects of DP have provided the supplement post-exercise, while, conversely, participants in studies using CH have consumed the supplement in varying doses in the days and weeks leading up to and, in some cases, also after damaging exercise [9,30,32,33,48,57]. Aussieker et al. [34] appear to be the exception, giving a single dose of CH post-exercise when investigating rates of protein synthesis. Therefore, although the timing used in this study is in line with the majority of studies investigating DP, it is possible that the supplementation period used in our study was insufficient and/or inappropriate to provide any benefit from CH.

It is unclear whether the positive effects CH has on indices of EIMD are the result of a protective effect provided by an increase in intramuscular collagen and/or due to an increased rate of cellular repair in the days after damaging exercise. The timing of our supplementation would only impact the latter, by providing substates to accelerate tissue repair and remodelling. Together with the lack of change in muscle connective protein synthesis reported by Aussieker et al. [34], our results suggest that, when only consumed after eccentric exercise, CH does not enhance recovery. As such, long term supplementation may be required in order to provide a protective effect to connective tissue, prior to eccentric exercise. This benefit was recently suggested by Bishof et al. [48], who found that supplementing concurrent training with CH over 12 weeks enhanced the repeated bout effect, reducing the level of damage occurring after a second bout of eccentric exercise. Additionally, Kuwaba et al. [57] reported that eccentric exercise was easier after 33 days of supplementation with CH. Although the reason for this is unclear, it is possible that adaptation to connective tissue, via an increase in hypoxia-inducible factor alpha 1 (HIF-α), heat shock proteins, and other initiators of collagen synthetic pathways [68], reduced stress on the musculotendinous system and enhanced force transmission and the SSC during repeated squats. As the majority of studies have only compared CH to a placebo [30,32,33,48,57], or they have lacked a placebo [9], it is unclear whether any protective effect is unique to CH or is provided by protein in general. The findings of Oikawa et al. [69] suggest that increases in intramuscular collagen are unaffected by CH or whey protein and, additionally, Rindom et al. [46] found that consuming whey protein or CH during a period of intensified training provided similar results on recovery; however, they used CH as their low-quality protein placebo and did not have a true control, so it is unclear if either protein was beneficial. Studies feeding DP in the days and weeks prior to eccentric exercise do not provide clarity, as the results are mixed [9,40,41,42].

It has been suggested that collagen may aid in the repair of the ECM through the delivery of non-essential amino acids glycine, proline, and hydroxyproline, and Pro-Hyp, which are believed to enhance collagen synthesis [70]. However, according to Prowting et al. [30], it is unclear whether the extra provision of non-essential amino acids is required to increase collagen synthesis or that it is possible for the body to sufficiently synthesise them without the need for an exogenous supply. Furthermore, despite CH-derived peptides appearing in circulation after ingestion [25], collagen supplementation does not enhance collagen synthesis after EIMD [9,30,32]. The inconsistent views on how CH effects collagen synthesis may stem from the difficulty identifying the synthesis of collagen in the skeletal muscle. It is difficult to accurately measure collagen synthesis without performing muscle biopsies [71] and, moreover, commonly used biomarkers of collagen synthesis are not specific to the synthesis of collagen in connective tissue, as they also reflect the synthesis of collagen in bone [30]. Clearly, standardised methods and more invasive measures may be required to fully understand the benefits and mechanisms of CH in the future.

As has been carried out by others [14,15,37,38,39,43], we used a combination of protein and carbohydrate for our treatments and matched this with an isoenergetic placebo. While carbohydrate may enhance the effect of protein by promoting the uptake of amino acids [72], it is a biologically active nutrient in its own right. Alone, carbohydrate has been shown to improve recovery by replenishing glycogen and providing energy for cellular recovery [15]. It can also contribute to anabolism in a similar way to the mechanistic actions of DP (e.g., by improving net protein balance [73]). Indeed, some studies have even found it to be more effective than dairy protein for recovery from EIMD [41,66]. As a result, we cannot rule out the effect that carbohydrate may have had alone and in combination with DP and CH; perhaps all treatments improved recovery equally.

As noted above, protein intervention studies are not without their limitations, and this study is no different. Although we screened participants based on their habitual dietary protein intake, we did not control this during the intervention period. Therefore, it is possible that participants consumed higher amounts of protein and, as such, protein from the treatments may not have had any benefit [74]. Additionally, we only recruited recreationally active male participants, and therefore it is unclear whether we would see the same results in individuals with different fitness levels and in females. Further, while downhill running induced significant changes in our measures, an exercise protocol that induces greater damage may provide a stronger stimulus for the interventions to interact with. Based on these limitations, and other gaps in our knowledge, we suggest that future research should control dietary protein intake and investigate different doses and combinations of proteins [75], different supplementation and exercise protocols, and the use of different populations. Additionally, there is a need to better understand the mechanisms responsible for the potential benefits of prolonged CH supplementation, which may require the use of more invasive measures than simply using the common measures of EIMD and changes in muscle function.

## 5. Conclusions

Our findings suggest that, when consumed for three days after damaging exercise, CH or DP does not expedite the rate of recovery any better than an isoenergetic placebo. Our findings add to the growing evidence that suggests that DP has little benefit for recovery from EIMD. For CH to have a beneficial effect on how muscle responds to eccentric exercise, consuming CH for a prolonged period, of at least nine days [32], prior to exercise may be necessary. We propose that individuals specifically wanting to speed up recovery after strenuous, damaging exercise should no longer consider acute, post-exercise protein supplementation as a reliable and valuable strategy.

## Figures and Tables

**Table 1:** Characteristics of participants in the dairy protein (DP), placebo (PLA), and collagen hydrolysate (CH) groups (mean (SD)). No differences were observed between groups.

	DP (*n* = 11)	PLA (*n* = 11)	CH (*n* = 11)	*p* Value
Age (years)	26.2 ± 7.2	23.5 ± 6.1	26.6 ± 6.4	0.468
Height (cm)	181.1 ± 11.4	182.0 ± 7.6	176.0 ± 5.3	0.220
Weight (kg)	87.0 ± 12.6	82.3 ± 13.1	82.6 ± 9.0	0.581
VO_2max_ (ml/kg/min)	45.8 ± 5.2	46.3 ± 5.3	45.2 ± 5.7	0.896
Protein (g/day) *	117.9 ± 55.8	103.7 ± 38.3	102.8 ± 38.4	0.697
Protein (g/day/kg BM) *	1.4 ± 0.6	1.3 ± 0.6	1.3 ± 0.5	0.905
Energy (MJ/day) *	10.8 ± 5.6	8.3 ± 2.1	10.0 ± 2.7	0.353

* Calculated from 24 h diet diaries recorded one week prior to familiarisation.

**Table 2:** Nutritional content of milk protein concentrate (DP), placebo (PLA), and collagen hydrolysate (CH) supplements.

Variable	DP	PLA	CH
Serving size (g)	35.8	53.2	26.9
Energy (kJ)	1000	1000	1000
Protein (g)	25.0	0.0	25.0
CHO (g)	32.9	58.8	33.8
Fat (g)	0.42	0.0	0.0

## Data Availability

Data are available from the corresponding author on request.

## Supplementary Materials

The following supporting information can be downloaded at: https://www.mdpi.com/article/10.3390/nu16244389/s1, Table S1: Amino acid profile of collagen hydrolysate (CH) and milk protein concentrate (DP).

## References

[1] Owens D.J., Twist C., Cobley J.N., Howatson G., Close G.L. (2019). Exercise-induced muscle damage: What is it, what causes it and what are the nutritional solutions?. Eur. J. Sport Sci..

[2] Bongiovanni T., Genovesi F., Nemmer M., Carling C., Alberti G., Howatson G. (2020). Nutritional interventions for reducing the signs and symptoms of exercise-induced muscle damage and accelerate recovery in athletes: Current knowledge, practical application and future perspectives. Eur. J. Appl. Physiol..

[3] Hody S., Croisier J.-L., Bury T., Rogister B., Leprince P. (2019). Eccentric muscle contractions: Risks and benefits. Front. Physiol..

[4] Tesarz J., Schuster A.K., Hartmann M., Gerhardt A., Eich W. (2012). Pain perception in athletes compared to normally active controls: A systematic review with meta-analysis. Pain.

[5] Aljamali N.M., Hussein K.M. (2021). Review on benefits and harms of nutritional supplements on health. J. Pharma Drug Regul. Aff..

[6] Peeling P., Binnie M.J., Goods P.S., Sim M., Burke L.M. (2018). Evidence-based supplements for the enhancement of athletic performance. Int. J. Sport Nutr. Exerc. Metab..

[7] Wang L., Meng Q., Su C.-H. (2024). From Food Supplements to Functional Foods: Emerging Perspectives on Post-Exercise Recovery Nutrition. Nutrients.

[8] O’Connor E., Mündel T., Barnes M.J. (2022). Nutritional compounds to improve post-exercise recovery. Nutrients.

[9] Robberechts R., Poffé C., Ampe N., Bogaerts S., Hespel P. (2023). Partly substituting whey for collagen peptide supplementation improves neither indices of muscle damage nor recovery of functional capacity during eccentric exercise training in fit males. Int. J. Sport Nutr. Exerc. Metab..

[10] West D.W., Abou Sawan S., Mazzulla M., Williamson E., Moore D.R. (2017). Whey protein supplementation enhances whole body protein metabolism and performance recovery after resistance exercise: A double-blind crossover study. Nutrients.

[11] Pavis G.F., Jameson T.S., Dirks M.L., Lee B.P., Abdelrahman D.R., Murton A.J., Porter C., Alamdari N., Mikus C.R., Wall B.T. (2021). Improved recovery from skeletal muscle damage is largely unexplained by myofibrillar protein synthesis or inflammatory and regenerative gene expression pathways. Am. J. Physiol..

[12] Brown M.A., Stevenson E.J., Howatson G. (2018). Whey protein hydrolysate supplementation accelerates recovery from exercise-induced muscle damage in females. Appl. Physiol. Nutr. Metab..

[13] Buckley J.D., Thomson R.L., Coates A.M., Howe P.R., DeNichilo M.O., Rowney M.K. (2010). Supplementation with a whey protein hydrolysate enhances recovery of muscle force-generating capacity following eccentric exercise. J. Sci. Med. Sport.

[14] Cooke M.B., Rybalka E., Stathis C.G., Cribb P.J., Hayes A. (2010). Whey protein isolate attenuates strength decline after eccentrically-induced muscle damage in healthy individuals. J. Int. Soc. Sports Nutr..

[15] Gee T.I., Woolrich T.J., Smith M.F. (2019). Effectiveness of whey protein hydrolysate and milk-based formulated drinks on recovery of strength and power following acute resistance exercise. J. Hum. Kinet..

[16] Nieman D.C., Zwetsloot K.A., Simonson A.J., Hoyle A.T., Wang X., Nelson H.K., Lefranc-Millot C., Guérin-Deremaux L. (2020). Effects of whey and pea protein supplementation on post-eccentric exercise muscle damage: A randomized trial. Nutrients.

[17] Ormsbee M.J., Saracino P.G., Morrissey M.C., Donaldson J., Rentería L.I., McKune A.J. (2022). Pre-sleep protein supplementation after an acute bout of evening resistance exercise does not improve next day performance or recovery in resistance trained men. J. Int. Soc. Sports Nutr..

[18] Saracino P.G., Saylor H.E., Hanna B.R., Hickner R.C., Kim J.-S., Ormsbee M.J. (2020). Effects of pre-sleep whey vs. plant-based protein consumption on muscle recovery following damaging morning exercise. Nutrients.

[19] Pearson A.G., Hind K., MacNaughton L.S. (2023). The impact of dietary protein supplementation on recovery from resistance exercise-induced muscle damage: A systematic review with meta-analysis. Eur. J. Clin. Nutr..

[20] Poore J., Nemecek T. (2018). Reducing food’s environmental impacts through producers and consumers. Science.

[21] Patel V., Aggarwal K., Dhawan A., Singh B., Shah P., Sawhney A., Jain R. (2024). Protein supplementation: The double-edged sword. Baylor University Medical Center Proceedings.

[22] Kurek M.A., Onopiuk A., Pogorzelska-Nowicka E., Szpicer A., Zalewska M., Półtorak A. (2022). Novel protein sources for applications in meat-alternative products—Insight and challenges. Foods.

[23] Holwerda A.M., van Loon L.J. (2022). The impact of collagen protein ingestion on musculoskeletal connective tissue remodeling: A narrative review. Nutr. Rev..

[24] Skov K., Oxfeldt M., Thøgersen R., Hansen M., Bertram H.C. (2019). Enzymatic hydrolysis of a collagen hydrolysate enhances postprandial absorption rate—A randomized controlled trial. Nutrients.

[25] Taga Y., Kusubata M., Ogawa-Goto K., Hattori S. (2014). Stable isotope-labeled collagen: A novel and versatile tool for quantitative collagen analyses using mass spectrometry. J. Proteome Res..

[26] Abe M., Hoshi T., Tajima A. (1987). Characteristics of transmural potential changes associated with the proton-peptide co-transport in toad small intestine. J. Physiol..

[27] Sato K., Jimi S., Kusubata M. (2019). Generation of bioactive prolyl-hydroxyproline (Pro-Hyp) by oral administration of collagen hydrolysate and degradation of endogenous collagen. Int. J. Food Sci. Technol..

[28] Sato K., Asai T.T., Jimi S. (2020). Collagen-derived di-peptide, prolylhydroxyproline (Pro-Hyp): A new low molecular weight growth-initiating factor for specific fibroblasts associated with wound healing. Front. Cell Dev. Biol..

[29] Tenberg S., Nosaka K., Wilke J. (2022). The relationship between acute exercise-induced changes in extramuscular connective tissue thickness and delayed onset muscle soreness in healthy participants: A randomized controlled crossover trial. Sports Med. -Open.

[30] Prowting J.L., Bemben D., Black C.D., Day E.A., Campbell J.A. (2020). Effects of collagen peptides on recovery following eccentric exercise in resistance-trained males—A pilot study. Int. J. Sport Nutr. Exerc. Metab..

[31] Wilke J., Behringer M. (2021). Is “delayed onset muscle soreness” a false friend? The potential implication of the fascial connective tissue in post-exercise discomfort. Int. J. Mol. Sci..

[32] Clifford T., Ventress M., Allerton D.M., Stansfield S., Tang J.C., Fraser W.D., Vanhoecke B., Prawitt J., Stevenson E. (2019). The effects of collagen peptides on muscle damage, inflammation and bone turnover following exercise: A randomized, controlled trial. Amino Acids.

[33] Lopez H.L., Ziegenfuss T.N., Park J. (2015). Evaluation of the effects of biocell collagen, a novel cartilage extract, on connective tissue support and functional recovery from exercise. Integr. Med. A Clin. J..

[34] Aussieker T., Hilkens L., Holwerda A.M., Fuchs C.J., Houben L.H., Senden J.M., Van Dijk J.-W., Snijders T., Van Loon L.J. (2023). Collagen protein ingestion during recovery from exercise does not increase muscle connective protein synthesis rates. Med. Sci. Sports Exerc..

[35] Bayles M.P. (2023). ACSM’s Exercise Testing and Prescription.

[36] Apweiler E., Wallace D., Stansfield S., Allerton D.M., Brown M.A., Stevenson E.J., Clifford T. (2018). Pre-bed casein protein supplementation does not enhance acute functional recovery in physically active males and females when exercise is performed in the morning. Sports.

[37] Betts J.A., Toone R.J., Stokes K.A., Thompson D. (2009). Systemic indices of skeletal muscle damage and recovery of muscle function after exercise: Effect of combined carbohydrate–protein ingestion. Appl. Physiol. Nutr. Metab..

[38] Cockburn E., Hayes P.R., French D.N., Stevenson E., St Clair Gibson A. (2008). Acute milk-based protein–CHO supplementation attenuates exercise-induced muscle damage. Appl. Physiol. Nutr. Metab..

[39] Cockburn E., Stevenson E., Hayes P.R., Robson-Ansley P., Howatson G. (2010). Effect of milk-based carbohydrate-protein supplement timing on the attenuation of exercise-induced muscle damage. Appl. Physiol. Nutr. Metab..

[40] Davies R.W., Bass J.J., Carson B.P., Norton C., Kozior M., Wilkinson D.J., Brook M.S., Atherton P.J., Smith K., Jakeman P.M. (2020). The effect of whey protein supplementation on myofibrillar protein synthesis and performance recovery in resistance-trained men. Nutrients.

[41] Hilkens L., Boerboom M., van Schijndel N., Bons J., van Loon L.J., van Dijk J.-W. (2023). Bone turnover following high-impact exercise is not modulated by collagen supplementation in young men: A randomized cross-over trial. Bone.

[42] Hirose N., Sato M., Yanagisawa O., Fukubayashi T. (2013). Milk peptide intake may decrease muscle damage after eccentric exercise. Int. J. Sport Health Sci..

[43] White J.P., Wilson J.M., Austin K.G., Greer B.K., St John N., Panton L.B. (2008). Effect of carbohydrate-protein supplement timing on acute exercise-induced muscle damage. J. Int. Soc. Sports Nutr..

[44] Markus I., Constantini K., Hoffman J., Bartolomei S., Gepner Y. (2021). Exercise-induced muscle damage: Mechanism, assessment and nutritional factors to accelerate recovery. Eur. J. Appl. Physiol..

[45] Lacroix M., Bos C., Léonil J., Airinei G., Luengo C., Daré S., Benamouzig R., Fouillet H., Fauquant J., Tomé D. (2006). Compared with casein or total milk protein, digestion of milk soluble proteins is too rapid to sustain the anabolic postprandial amino acid requirement. Am. J. Clin. Nutr..

[46] Rindom E., Nielsen M., Kececi K., Jensen M., Vissing K., Farup J. (2016). Effect of protein quality on recovery after intense resistance training. Eur. J. Appl. Physiol..

[47] Draganidis D., Chondrogianni N., Chatzinikolaou A., Terzis G., Karagounis L.G., Sovatzidis A., Avloniti A., Lefaki M., Protopapa M., Deli C.K. (2017). Protein ingestion preserves proteasome activity during intense aseptic inflammation and facilitates skeletal muscle recovery in humans. Br. J. Nutr..

[48] Bischof K., Stafilidis S., Bundschuh L., Oesser S., Baca A., König D. (2024). Reduction in systemic muscle stress markers after exercise-induced muscle damage following concurrent training and supplementation with specific collagen peptides–a randomized controlled trial. Front. Nutr..

[49] Etheridge T., Philp A., Watt P.W. (2008). A single protein meal increases recovery of muscle function following an acute eccentric exercise bout. Appl. Physiol. Nutr. Metab..

[50] Hyldahl R.D., Chen T.C., Nosaka K. (2017). Mechanisms and mediators of the skeletal muscle repeated bout effect. Exerc. Sport Sci. Rev..

[51] Bontemps B., Vercruyssen F., Gruet M., Louis J. (2020). Downhill running: What are the effects and how can we adapt? A narrative review. Sports Med..

[52] Chen T.C., Nosaka K., Tu J.-H. (2007). Changes in running economy following downhill running. J. Sports Sci..

[53] Chrismas B.C., Taylor L., Siegler J.C., Midgley A.W. (2017). A reduction in maximal incremental exercise test duration 48 h post downhill run is associated with muscle damage derived exercise induced pain. Front. Physiol..

[54] Haff G.G., Stone M., O’Bryant H.S., Harman E., Dinan C., Johnson R., Han K.-H. (1997). Force-time dependent characteristics of dynamic and isometric muscle actions. J. Strength Cond. Res..

[55] Beckham G., Mizuguchi S., Carter C., Sato K., Ramsey M., Lamont H., Hornsby G., Haff G., Stone M. (2013). Relationships of isometric mid-thigh pull variables to weightlifting performance. J. Sports Med. Phys. Fit..

[56] Barnes M.J., Mündel T., Stannard S.R. (2012). The effects of acute alcohol consumption and eccentric muscle damage on neuromuscular function. Appl. Physiol. Nutr. Metab..

[57] Kuwaba K., Kusubata M., Taga Y., Igarashi H., Nakazato K., Mizuno K. (2023). Dietary collagen peptides alleviate exercise-induced muscle soreness in healthy middle-aged males: A randomized double-blinded crossover clinical trial. J. Int. Soc. Sports Nutr..

[58] Warren G.L., Lowe D.A., Armstrong R.B. (1999). Measurement tools used in the study of eccentric contraction-induced injury. Sports Med..

[59] Brancaccio P., Maffulli N., Limongelli F.M. (2007). Creatine kinase monitoring in sport medicine. Br. Med. Bull..

[60] Paulsen G., Ramer Mikkelsen U., Raastad T., Peake J.M. (2012). Leucocytes, cytokines and satellite cells: What role do they play in muscle damage and regeneration following eccentric exercise?. Exerc. Immunol. Rev..

[61] Nash D., Hughes M.G., Butcher L., Aicheler R., Smith P., Cullen T., Webb R. (2023). IL-6 signaling in acute exercise and chronic training: Potential consequences for health and athletic performance. Scand. J. Med. Sci. Sports.

[62] León-López A., Fuentes-Jiménez L., Hernández-Fuentes A.D., Campos-Montiel R.G., Aguirre-Álvarez G. (2019). Hydrolysed collagen from sheepskins as a source of functional peptides with antioxidant activity. Int. J. Mol. Sci..

[63] Burnley E.C.D., Olson A.N., Sharp R.L., Baier S.M., Alekel D.L. (2010). Impact of protein supplements on muscle recovery after exercise-induced muscle soreness. J. Exerc. Sci. Fit..

[64] Dahlstrom E.C. (2007). Impact of Protein Supplementation on Muscle Recovery After Exercise-Induced Muscle Soreness.

[65] Eddens L., Browne S., Stevenson E.J., Sanderson B., van Someren K., Howatson G. (2017). The efficacy of protein supplementation during recovery from muscle-damaging concurrent exercise. Appl. Physiol. Nutr. Metab..

[66] Ten Haaf D.S., Flipsen M.A., Horstman A.M., Timmerman H., Steegers M.A., De Groot L.C., Eijsvogels T.M., Hopman M.T. (2021). The effect of protein supplementation versus carbohydrate supplementation on muscle damage markers and soreness following a 15-km road race: A double-blind randomized controlled trial. Nutrients.

[67] Pasiakos S.M., Lieberman H.R., McLellan T.M. (2014). Effects of protein supplements on muscle damage, soreness and recovery of muscle function and physical performance: A systematic review. Sports Med..

[68] Oertzen-Hagemann V., Kirmse M., Eggers B., Pfeiffer K., Marcus K., de Marées M., Platen P. (2019). Effects of 12 weeks of hypertrophy resistance exercise training combined with collagen peptide supplementation on the skeletal muscle proteome in recreationally active men. Nutrients.

[69] Oikawa S.Y., Kamal M.J., Webb E.K., McGlory C., Baker S.K., Phillips S.M. (2020). Whey protein but not collagen peptides stimulate acute and longer-term muscle protein synthesis with and without resistance exercise in healthy older women: A randomized controlled trial. Am. J. Clin. Nutr..

[70] Shaw G., Lee-Barthel A., Ross M.L., Wang B., Baar K. (2017). Vitamin C–enriched gelatin supplementation before intermittent activity augments collagen synthesis. Am. J. Clin. Nutr..

[71] Kviatkovsky S.A., Hickner R.C., Ormsbee M.J. (2022). Collagen peptide supplementation for pain and function: Is it effective?. Curr. Opin. Clin. Nutr. Metab. Care.

[72] Starkoff B.E., Lenz E.K., Mattern C.O., Too D., Byrne H.K. (2020). Protein Supplementation Does Not Enhance Recovery from Exercise-Induced Muscle Damage. J. Exerc. Physiol. Online.

[73] Børsheim E., Cree M.G., Tipton K.D., Elliott T.A., Aarsland A., Wolfe R.R. (2004). Effect of carbohydrate intake on net muscle protein synthesis during recovery from resistance exercise. J. Appl. Physiol..

[74] Schoenfeld B.J., Aragon A.A. (2018). How much protein can the body use in a single meal for muscle-building? Implications for daily protein distribution. J. Int. Soc. Sports Nutr..

[75] Deane C.S., Bass J.J., Crossland H., Phillips B.E., Atherton P.J. (2020). Animal, plant, collagen and blended dietary proteins: Effects on musculoskeletal outcomes. Nutrients.

