# Supplementary material for: Comparing the Effects of Collagen Hydrolysate and Dairy Protein on Recovery from Eccentric Exercise: A Double Blind, Placebo-Controlled Study
Source: Nutrients. 2024 Dec 20;16(24):4389. doi: 10.3390/nu16244389 (PMC11678417; doi:10.3390/nu16244389)
Supplement: Supplementary file 1 [file nutrients-16-04389-s001.zip › nutrients-3338163-supplementary.pdf]

Table S1. Amino acid profile of dairy protein (DP) collagen hydrolysate (CH)

Amino Acid (mg/100 mg)	DP	CH
Aspartic Acid	78	5.95
Threonine	45	1.74
Serine	56	3.03
Glutamic Acid	216	8.12
Proline	98	12.75
Glycine	19	22.68
Alanine	33	8.57
Tryptophan	14	-
Valine	64	2.72
Methionine	28	0.91
Isoleucine	53	1.51
Leucine	96	2.95
Tyrosine	53	1.23
Phenylalanine	49	2.14
Histidine	27	0.68
Lysine	84	4.08
Arginine	37	8.18
Hydroxyproline	-	10.79
Cysteine	12	0.02
